# Supplementary material for: Italian Validation of the Short Version of the Failure to Mentalize Trauma Questionnaire in Adults at Risk Due to Childhood Trauma
Source: Behav Sci (Basel). 2023 Oct 15;13(10):843. doi: 10.3390/bs13100843 (PMC10604537; doi:10.3390/bs13100843)
Supplement: Supplementary file 1 [file behavsci-13-00843-s001.zip › behavsci-2622979-supplementary.pdf]

Items excluded from the FMTQ-s:

1) People tell me I minimize difficult experiences.
2) I was treated badly but I deserved it.
3) When I talk about difficult experiences I have lived, I realize I give a lot of attention to details, such as the places, the smells, the sounds.
6) I am surprised to notice how sensitive I can be in general, but how cold I am when facing dramatic situations.
15) People who were mean to me were well-intended.
16) Many people would have been traumatized if they were in my place, but I was strong enough to overcome the difficult experiences I have lived.
17) There is nothing else like violence (ex. fighting, arguing with someone, watching violent movies) to vent negative feelings.
19) Certain people like me are strong enough to overcome efficiently difficult life experiences alone and without any help.
20) The best way to face the difficult experiences I have lived is to avoid thinking about it.
21) I tend to be aggressive toward others because it helps to blow off steam.
22) Facing difficult life experiences has made me stronger. I am now ready to deal with anything.
24) I feel comfortable in relationships where there are open conflicts or some fights.
25) I have the strength to cut myself off from undesirable emotions associated with difficult experiences I have lived, as if they never happened.
27) Seeing other people suffer makes me feel better.
28) If I had been in the shoes of the people that hurt me, I would have acted in the same way.
29) People who are abusive or violent are mainly unconscious: they don’t really want to hurt people.
